# Feasibility evaluation of a virtual lifestyle intervention for early-stage breast cancer survivors undergoing chemotherapy

**DOI:** 10.1093/jncics/pkaf122

**Published:** 2026-01-31

**Authors:** Sim Yee (Cindy) Tan, Isaac Yeboah Addo, Gemma Collett, Emily Price, Eliza R Macdonald, Shannon Gerber, Jane Turner, Liane Lee, Hau Yi Yau, Jaclyn Spencer, Sama Saleem, Antonia Pearson, Frances Boyle, Stephen Della-Fiorentina, Belinda E Kiely, Natalie Taylor, Jasmine Yee, Richard De Abreu Lourenco, Adrian Bauman, Haryana M Dhillon, Janette L Vardy

**Affiliations:** Concord Cancer Centre, Concord Repatriation General Hospital, NSW, Australia; Faculty of Health and Medicine, University of Sydney, Sydney, NSW, Australia; Nutrition and Dietetics Department, Concord Hospital, Concord, NSW, Australia; Faculty of Health and Medicine, University of Sydney, Sydney, NSW, Australia; Faculty of Health and Medicine, University of Sydney, Sydney, NSW, Australia; Psycho-Oncology Co-operative Research Group, School of Psychology, University of Sydney, Sydney, NSW, Australia; Concord Cancer Centre, Concord Repatriation General Hospital, NSW, Australia; School of Health Sciences, Faculty of Medicine and Health, University of New South Wales, Sydney, NSW, Australia; Concord Cancer Centre, Concord Repatriation General Hospital, NSW, Australia; Concord Cancer Centre, Concord Repatriation General Hospital, NSW, Australia; Faculty of Health and Medicine, University of Sydney, Sydney, NSW, Australia; Faculty of Health and Medicine, University of Sydney, Sydney, NSW, Australia; Nutrition and Dietetics Department, Concord Hospital, Concord, NSW, Australia; Nutrition and Dietetics Department, Concord Hospital, Concord, NSW, Australia; Northern Beaches Hospital, Frenchs Forest, Sydney, NSW, Australia; Patricia Ritchie Centre for Cancer Care and Research, Mater Hospital, Sydney, NSW, Australia; Southern Highlands Cancer Centre, Bowral, NSW, Australia; Concord Cancer Centre, Concord Repatriation General Hospital, NSW, Australia; Implementation to Impact, School of Population Health, Faculty of Medicine and Health, University of New South Wales, Sydney, NSW, Australia; Faculty of Health and Medicine, University of Sydney, Sydney, NSW, Australia; Psycho-Oncology Co-operative Research Group, School of Psychology, University of Sydney, Sydney, NSW, Australia; Centre for Health Economics Research and Evaluation, University of Technology, Sydney, NSW, Australia; School of Public Health, University of Sydney, Sydney, NSW, Australia; Psycho-Oncology Co-operative Research Group, School of Psychology, University of Sydney, Sydney, NSW, Australia; Concord Cancer Centre, Concord Repatriation General Hospital, NSW, Australia; Faculty of Health and Medicine, University of Sydney, Sydney, NSW, Australia

## Abstract

**Background:**

Weight gain and physical inactivity during chemotherapy for patients with early-stage breast cancer are common. We sought to investigate the feasibility of a virtual lifestyle (exercise and diet) intervention for breast cancer survivors during chemotherapy.

**Methods:**

This single-arm phase 2 trial delivered 12 weekly 1-hour telehealth sessions of supervised exercise and diet education to breast cancer survivors (patients with stage I-III disease) starting neoadjuvant chemotherapy. Screening, recruitment, intervention, and study assessments completed at baseline (T0), immediately after the intervention (T1), and 3 months after the intervention (T2) were conducted by telehealth in 2022-2023. The primary outcome was that at least 60% of participants achieved 50% of the predetermined exercise and dietary goals. Secondary outcomes were acceptability (participation, attendance, completion), physical health, and lifestyle outcomes.

**Results:**

Of 73 referrals, 60 individuals were eligible, 58 (97%) provided consent, 51 (85%) commenced the intervention, and 34 (57%) completed at least 1 postintervention assessment (completion rate = 67%). The mean (SD) age of participants was 51 (8.8) years, and 50% of participants were receiving neoadjuvant chemotherapy. Attendance was lower for exercise than for diet sessions (44% vs 62% attended ≥75% sessions). At T1, 36% of participants adhered to at at least 50% of the preset goals, improving at T2 (62.5%). Weight was not statistically significantly different between T0 and T1 (*P* = .199) but increased substantially at T2 (*P* = .018). Average waist circumference was reduced at T1 (‒1.9 cm, *P* = .014) and at T2 (‒3.3 cm, *P* < .001). Weekly exercise time increased by 38.5 minutes from T0 to T1 (*P* = .038), and the proportion of participants who met exercise guidelines improved from 6% (T0) to 41% (T2).

**Conclusion:**

Our primary outcome was not achieved immediately after the intervention but was observed 3 months later. Individuals completing the intervention attended at least half the diet and exercise sessions during chemotherapy. Results of this study will inform design of a phase 3 study.

## Introduction

More than 50% of breast cancer survivors gain weight after primary treatment, have overweight or obesity, and are physically inactive during treatment.^1^ Obesity and physical inactivity are modifiable lifestyle factors associated with cancer risk and recurrence.^2^ Studies indicate a 12% increased risk of breast cancer (postmenopausal) and a 20% increase in breast cancer–specific mortality for every 5 kg/m^2^ body mass index (BMI) increment.^3^

Our research revealed that 63% (101/160) of breast cancer survivors attending their initial Sydney Cancer Survivorship Centre clinic visit had overweight or obesity approximately 11 months after their diagnosis^4^; only 33% of breast cancer survivors met aerobic exercise recommendations, and none adhered to both resistance and aerobic exercise recommendations.^5^

Among breast cancer survivors, physical activity declines during treatment.^6^ Exercise can ameliorate side effects associated with chemotherapy, improving fatigue, physical function, psychosocial distress, and health-related quality of life (QOL).^7-10^ Women who engage in the highest levels of physical activity following a breast cancer diagnosis experience substantially lower cancer-specific and all-cause mortality (37% and 42% lower risk, respectively) compared with the least active patients.^11^ Epidemiological evidence shows that physical activity improves survival in people with cancer.^12^ The recent randomized Phase III Study of the Impact of a Physical Activity Program on Disease-Free Survival in Patients With High-Risk Stage II or Stage III Colon Cancer: A Randomized Controlled Trial (CHALLENGE; ClinicalTrials.gov identifier NCT00819208) demonstrated that physical activity protects against cancer recurrence and reduces mortality in colon cancer survivors.^13^ Furthermore, maintaining a healthy lifestyle, including regular exercise and healthy eating, can improve physical health and long-term well-being.^14^^,^^15^ The World Cancer Research Fund (WCRF) recommends that breast cancer survivors (1) be physically active, increasing exercise under supervision; (2) increase dietary fiber intake; and (3) avoid unnecessary weight gain during or after treatment.^3^

The Women’s Health Initiative dietary modification randomized trial demonstrated that a low-fat diet, with increased intake of fruit, vegetables, and legumes, had long-term benefits for health outcomes and breast cancer–related mortality.^16^ Healthy eating patterns improve overall health and reduce cancer risk.^17^ A Cochrane review of 23 interventions targeting breast cancer survivors with overweight or obesity found that combining diet with exercise or psychosocial support had a greater impact on body weight and waist circumference than did dietary interventions alone.^18^

Exercise in advanced and early-stage cancer settings is feasible and safe during chemotherapy.^19-22^ Despite evidence of health benefits and cancer risk reduction as well as recommendations from international cancer organisations,^3^^,^^23^ the majority (73%) of lifestyle intervention trials in breast cancer survivors were conducted after completion of chemotherapy.^24^ Evidence for breast cancer survivor lifestyle interventions delivered during treatment is limited.^25^^,^^26^

Due to limited staff resources, public hospital supportive services (eg, dietetics, physiotherapy) often focus on treating patients with acute symptoms during treatment rather than supporting long-term lifestyle changes, which are resource intensive and take time to deliver changes in health behaviors. A structured, multimodal lifestyle intervention involving diet and exercise delivered through an online platform may provide a flexible approach to help breast cancer survivors improve or maintain a healthy lifestyle during chemotherapy. Virtual programs may provide a person-centered approach to increase equitable, accessible care that is scalable. It may overcome barriers such as distance, parking, infection risk, and attending when feeling unwell and be cost-effective.^27^

We evaluated the feasibility of delivering a multimodal, structured telehealth **L**ifestyle intervention (**E**xercise **A**nd **D**iet) for **B**reast **C**ancer **S**urvivors (LEAD-4-BCS) during adjuvant or neoadjuvant chemotherapy. We hypothesized that LEAD-4-BCS would be feasible and acceptable and would help breast cancer survivors maintain or improve healthy lifestyle behaviors during chemotherapy.

## Methods

### Study design

LEAD-4-BCS was a phase 2 multisite feasibility study evaluating 12 weekly structured, supervised exercise and diet education sessions delivered using a virtual platform. Ethics approval was granted by the Sydney Local Health District, Concord Repatriation General Hospital Human Research Ethics Committee (CH62/6/2022-031). All participants provided informed consent. The study was registered with the Australian New Zealand Clinical Trials Registry (ACTRN12622000741785).

### Eligibility

Participants were at least 18 years old, had a diagnosis of early-stage breast cancer (stages I-III), and had commenced neoadjuvant or adjuvant chemotherapy, with at least 12 weeks of chemotherapy remaining. Inclusion required no comorbidities that would limit exercise (eg, uncontrolled hypertension) and access to the virtual platform Zoom (Zoom Communications, Inc). Medical clearance was required if concerns were identified on exercise and falls risk screening questions. Participants who were at risk of falls; unable to provide informed consent; on a medical diet that contradicted high-fiber, low-fat dietary recommendations; visually impaired; had a concurrent illness that required high-dose steroids (eg, 25 mg prednisone); or had comorbidities that could potentially hamper adherence were excluded.

### Screening and recruitment

**Figure 1.**
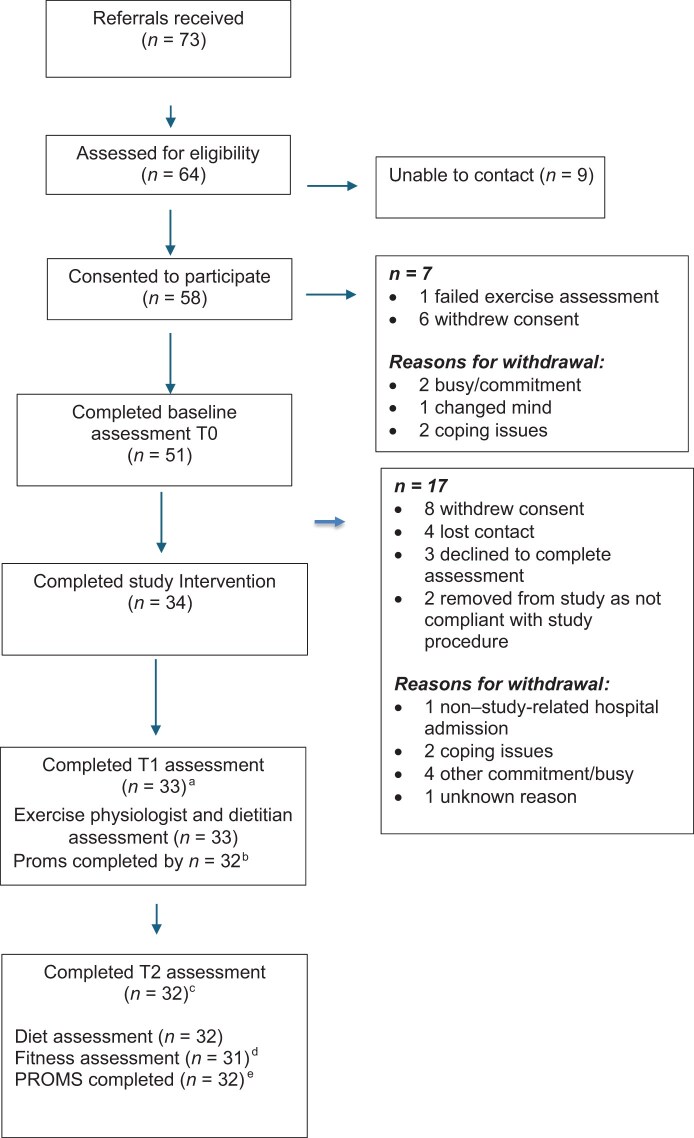
CONSORT diagram of participants in the study. T0 = baseline; T1 = immediately after intervention; T2 = 3 months after intervention. ^a^One patient unable to complete the T1 assessment because her surgery was brought forward. ^b^One patient did not complete patient-reported outcomes measures (PROMs). ^c^Two patients were lost to follow-up, but one patient who missed the T1 assessment returned for the T2 assessment. ^d^One patient was missing a fitness assessment because of a recent surgery.

Potential participants at 11 Australian sites were approached by their treating team to gauge interest. The central study coordinator subsequently contacted the patients for screening and consent via telehealth. Figure 1 shows the CONSORT diagram.

### Study intervention

The intervention lasted up to 14 weeks and consisted of a 1-hour diet education session and a 1-hour supervised exercise session weekly for the first 10 weeks. To support transition to self-management, the final 2 sessions could be scheduled 2 weeks apart. Sessions were delivered online by an experienced, accredited dietitian and exercise physiologist. Individualized dietary and exercise recommendations were provided at baseline.

#### Diet

Dietary education sessions covered topics aligned with healthy eating for cancer prevention and dietary recommendations focused on improving diet quality and lifestyle behaviors, such as meeting Australian dietary guidelines for fruit (2 servings per day) and vegetables (5 servings per day), minimizing discretionary foods^28^ rather than caloric restrictions. Participants were asked to keep a daily diary, recording total consumption of fruit and vegetables,^29^ and a 24-hour diet recall once a week to encourage self-reflection. Sessions included peer support, where participants could share their thoughts, concerns, and questions in discussions the study dietitian facilitated.

#### Exercise

Individualized weekly exercise recommendations included a combination of moderate to vigorous aerobic (eg, walking, cycling) and resistance (eg, resistance bands, weights) training.^5^ The 12 structured exercise sessions included circuit-based aerobic exercises (20 minutes); resistance exercises (30 minutes) targeting major muscle groups; and behavior change support, such as behavior feedback. Exercises were tailored to individual capacity.

Text messages with encouraging statements (sourced from a published study)^30^ and investigator-developed prompts were sent daily to support adherence to a healthy lifestyle (see Supplementary Methods). Resources from reputable organizations (eg, Cancer Council Australia) were provided as required to enforce healthy diet and exercise messages.

### Outcome assessments

Participants were assessed at baseline (T0), immediately after the intervention (T1), and 3 months after the intervention (T2). Participants were considered “completers” if they had completed the study intervention and at least 1 of the postintervention assessments (T1 or T2). All assessments were conducted over Zoom.

### Primary outcome

If at least 60% of study participants could achieve 50% of their preset dietary or exercise goals, this virtual lifestyle intervention would be deemed feasible for promoting a healthy lifestyle among breast cancer survivors during their chemotherapy treatment. Participants set their own exercise and dietary goals at T0, with guidance from the dietitian and exercise physiologist. These goals were used to measure individual adherence at T1. The personal goals focused on daily fruit and vegetable intake as well as weekly exercise levels, working toward the Australian dietary guidelines^28^ and Clinical Oncology Society of Australia exercise guidelines.^5^ We adopted a pragmatic approach, encouraging participants to set realistic dietary and exercise goals, especially during this challenging period. See Table 1 for a list of predetermined dietary and exercise goals. Participants were considered adherent if they met 50% of their preset dietary or exercise goals.

**Table 1.** List of dietary and exercise goals set by study participants who had completed the study intervention (*n* = 34).

Goals for fruit and vegetable intake based on the Australian dietary guidelines (2 servings of fruit and 5 servings of vegetable per day)	Exercise goals based on the Clinical Oncology Society of Australia Exercise Position Statement (150 min of moderate-intensity or 75 min of vigorous-intensity exercise and 2 sessions of resistance training per week)
Dietary goals set by study participants Fruit: Aim for 2 servings of fruit/d (*n* = 31); and 1 serving of fruit/d (*n* = 1).	Exercise goals set by participants Aerobic exercise: Aim for 150 min of moderate-intensity aerobic exercise/wk (*n* = 26); 120 min of moderate-intensity aerobic exercise/wk (*n* = 2); 100 min of moderate-intensity aerobic exercise/wk (*n* = 2); 90 min of moderate-intensity aerobic exercise/wk (*n* = 1); and 100 min of aerobic exercise/wk (no specific exercise intensity) (*n* = 1).
Reduce fruit intake to 2-3 servings/d (*n* = 2)	Maintain 150 min of moderate-intensity to vigorous-intensity aerobic exercise/wk (*n* = 2)
Vegetables: Aim for 5 servings of vegetables/d (*n* = 27); 4.5 servings of vegetables/d (*n* = 1); 4 servings of vegetables/d (*n* = 3); and 3 servings of vegetables/d (*n* = 3).	Resistance training: Aim for 2 sessions/wk (*n* = 31); and 1 session/wk (*n* = 3).
**Additional goals (optional and not part of adherence measurement)**	
Better snack options (*n* = 1) Reduce snacks (*n* = 2)	Daily stretching (*n* = 1) 2 stretching sessions/wk (*n* = 2)
Have regular meals (*n* = 1)	Gardening 3-4 times/wk (*n* = 1)
Reduce alcohol intake (*n* = 2) ≤5 standard alcohol drinks/wk (*n* = 1)	Attend social netball session when able to (*n* = 1) and Pilates when able to (*n* = 1)
Increase protein and iron-rich food intake (*n* = 1)	

### Secondary outcomes

Participants’ acceptability (participation, study completion, attendance rate, and qualitative interview data) and lifestyle outcomes (anthropometry, diet, physical activity, adherence to lifestyle recommendations for cancer prevention from the WCRF,^31^ exercise assessments, and QOL) were secondary outcomes. Descriptions of outcome measures are available in the Supplementary Methods.

### Sample size

The planned sample size was 50 participants, allowing for 20% attrition. Feasibility of the intervention was defined as at least 60% of participants adhering to at least half of the preset exercise or diet goals.^32^

### Statistical analyses

Baseline characteristics were analyzed descriptively, with results reported as mean (SD) or median (IQR). Parametric *t* tests and analysis of variance were used to compare continuous variables between assessment time points, and nonparametric tests such as Mann-Whitney *U* and Wilcoxon signed rank were used for non-normally distributed data. The McNemar test was applied for within-group comparisons, and the χ^2^ test was applied for between-group comparisons. IBM SPSS, version 29, software was used to conduct the quantitative analysis. *P* < .05 was considered statistically significant.

A norm-based *t* score, calculated based on the mean raw scores from the European Organisation for Research and Treatment of Cancer QLG Core Questionnaire assessment tool, was used to compare the Australian general population with the Australian population with cancer. The norm-based *t* score was generated using a calculation tool from the Cancer Quality of Life Expert Service Team.^33^

Qualitative data from the semistructured exit interviews were analyzed using the Framework method,^34^ an inductive approach, to explore the data in the context of Temporal Self-Regulation Theory.^35^ The following strategies were used to ensure methodological rigor: iterative revision of the interview guide, creation of interviewer memos, transcription review, verbal debriefing, use of participant quotations, and multiple codes and cross-coding.^36^

## Results

Table 2 presents baseline characteristics of the 51 female participants who completed the T0 assessment; comparing women who completed the intervention and at least 1 poststudy assessment (completers; *n* = 34) with women who did not (noncompleters; *n* = 17). Mean (SD) age was 51 (9.8) years; all women were slated to undergo chemotherapy (51% neoadjuvant chemotherapy), and 22 (43%) were already receiving chemotherapy at enrolment.

**Table 2.** Demographics and baseline comparison of participants who completed at least 1 postintervention assessment (completers) and who did not (noncompleters).

Variable	**All** **(*n* = 51)**	**Completers** **(*n* = 34)**	**Noncompleters** **(*n* = 17)**	*P* (2-sided)
Age, mean (SD), y	51.25 (9.8)	50.9 (8.8)	51.9 (11.7)	.73
Aged ≥65 y, No. (%)	4 (8)	2 (6)	2 (12)	.60
Level of education completed, No. (%)				
Year ≤12	10 (20)	5 (15)	5 (29)	.27
Technical and Further Education, undergraduate or postgraduate	41 (80)	29 (85)	12 (71)	
Work status, No. (%)				
Currently working (full or part time)	24 (53)	17 (50)	7 (41)	.77
Currently not working (retired, on sick leave, or other^a^)	27 (47)	17 (50)	10 (59)	
Marital status, No. (%)				
Married/de facto	40 (78)	27 (79)	13 (77)	>.99
Other—separated, single, or widowed	11 (22)	7 (21)	4 (24)	
Stage of disease, No. (%)				
1	10 (20)	6 (18)	4 (24)	.31
2	28 (55)	17 (50)	11 (65)	
3	13 (26)	11 (32)	2 (12)	
Receiving chemotherapy at the time of enrollment, No. (%)				
Yes	22 (43)	13 (38)	9 (53)	.38
No	29 (57)	21 (62)	8 (47)	
Receiving neoadjuvant chemotherapy, No. (%)	26 (51)	17 (50)	9 (53)	>.99
Receiving or about to start adjuvant chemotherapy, No. (%)	25 (49)	17 (50)	8 (47)	
Had surgery at the time of enrollment, No. (%)				
Yes	25 (49)	17 (50)	8 (47)	>.99
Mastectomy	9 (36)	6 (35)	3 (37.5)	
Lumpectomy	16 (64)	11 (65)	5 (62.5)	
Comorbidities, No. (%)				
No comorbidities reported	19 (37)	15 (44)	4 (24)	.22
Diabetes	2 (4)	1 (3)	1 (6)	NA
Hypercholesterolemia	4 (8)	2 (6)	2 (12)	NA
Hypertension	6 (12)	4 (12)	2 (12)	NA
Anxiety and/or depression	6 (12)	5 (15)	1 (6)	NA
Smoking history, No. (%)				
Never	30 (59)	23 (68)	7 (41)	.07
Former smoker	20 (39)	11 (32)	9 (53)	
Current	1 (2)	0 (0)	1 (6)	
Current alcohol consumption, No. (%)				
No	35 (69)	19 (56)	16 (94)	.009
Yes	16 (31)	15 (44)	1 (6)	
Anthropometry				
Body weight, mean (SD), kg	76.5 (18.6)	72.0 (16.43)	85.5 (19.8)	.02^b^
BMI, mean (SD), kg/m^2^	28.0 (6.3)	26.5 (5.7)	30.8 (6.6)	.01^b^
19.5-24.5 (normal)	20 (39)	16 (47)	4 (24)	.10
25-29.9 (overweight)	14 (28)	10 (29)	4 (24)	
≥30 (obese)	17 (33)	8 (24)	9 (53)	
Waist circumference, mean (SD), cm	*n* = 48	*n* = 32	*n* = 16	.003
	92.3 (15.9)	87.6 (14.7)	101.5 (14.4)	

Abbreviation: BMI = body mass index; NA = not assessed.

a “Other” included student, carer role, career break, and looking for work.

b Data not normally distributed; nonparametric tests used for comparisons.

There was no statistically significant age difference between completers and noncompleters (*P* = .73). Noncompleters had statistically significantly higher baseline body weight (*P* = .02), BMI (*P* = .01), and waist circumference (*P* = .003).

### Acceptability: uptake, study completion, and attendance rate

Seventy-three referrals were received from 11 sites over 7.5 months. Of 73 people referred, 64 (88%) completed screening; of those 64 women, 60 (94%) were eligible. The consent rate was 97% (58/60), and the commencement rate was 85% (51/60). Thirty-four participants completed the study, resulting in a study completion rate of 67% (34/51) (Figure 1).

Among the 34 completers, the median attendance rate for supervised exercise and diet education sessions was 75% (IQR = 53.6%-91.4%). Fifteen participants (44%) attended 75% of available exercise sessions, while 21 (62%) attended 75% of available diet education sessions.

Thirty-five exit interviews were conducted: 33 by study completers and 2 by noncompleters. We identified 4 themes: self-awareness, psychosocial impacts, feasible integration into daily routine, and treatment-related obstacles. Interviewees perceived the intervention as feasible to integrate into daily routines because it was flexible; online delivery overcame accessibility concerns, and the structured approach reinforced consistency in activity and diet. Obstacles included fluctuations in chemotherapy side effects and perceived relevance of health advice. The program was perceived to increase confidence in adopting healthier lifestyle behaviors after the intervention, but lack of external accountability after the supervised program ended was a concern for some participants.

### Adherence rate

Among participants who completed the T1 assessment, 36% (12/33) achieved at least 50% of their preset dietary and exercise goals, and 58% (19/33) achieved at least one-quarter of preset goals. Consequently, the intervention feasibility target (60%) was not met at T1. By T2, adherence to at least 50% of the preset goals increased to 63% (20/32), exceeding the feasibility cutoff (Table 3).

**Table 3.** Adherence rate to preset diet or exercise goals at the postintervention and 3-month follow-up assessments.

	**Post-study assessment, No. (%)** **(*n* = 33)**	**3-mo follow-up assessment, No. (%)** **(*n* = 32)**
Adhered to at least 50% of the dietary or exercise preset goals	12 (36.4)	20 (62.5)
≥50% of preset exercise goals only	14 (42.5)	20 (62.5)
≥50% of preset dietary goals only	15 (45.5)	17 (53.1)

### Effect on lifestyle outcomes

#### Diet

**Figure 2.**
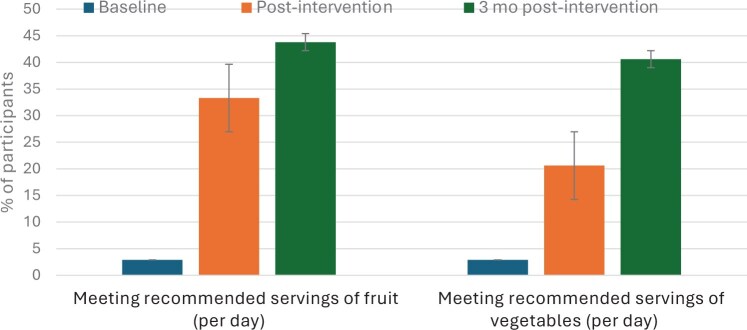
Proportion of participants meeting the Australian dietary guidelines–recommended servings of fruit and vegetables per day at baseline (T0), immediate postintervention (T1), and 3-month postintervention (T2) assessments. Paired data were used for comparison between time points (T1-T0, T2-T0).

Fruit and vegetable intake improved between T0 and T1; fruit intake improved from a median 1.1 (IQR = 0.80-1.8) to 1.9 (IQR = 1.1-2.2) servings per day (*P* = .003), and vegetable intake improved from a mean (SD) 2.7 (1.1) to 3.6 (1.5) servings per day (*P* = .004). This improvement was sustained at T2, with fruit intake remaining at a median 1.9 (IQR = 1.1-2.2) servings per day and vegetables a mean (SD) 3.8 (1.7) servings per day. Figure 2 shows an increase in the proportion of participants meeting the Australian dietary guidelines–recommended 2 servings of fruit and 5 servings of vegetables per day from T0 to T2.

Three-day food diaries revealed a nonsignificant trend for energy intake to be higher at the T1 assessment (mean difference = 236 kJ/day, 95% CI = 430.2 to 901.6) and a statistically significant improvement in fiber intake (mean difference = 3.0 g/d, 95% CI = 0.8 to 5.3), fruit intake (median difference = 65 g/d [IQR = ‒1.1 to 2180.4 g], *P* < .001), and vegetable intake (mean difference = 62.3 g/d, 95% CI = 4.4 to 120.2). These differences in fruit and vegetable intake were maintained at T2.

The average (SD) WCRF score for 29 participants was 3.9 (1.4) at T0, 4.3 (1.2) at T1, and 4.6 (1.4) at T2. Post hoc analysis showed a statistically significant improvement in WCRF score between T0 and T2 (0.75 points, 95% CI = 0.174 to 1.33) but no difference between T0 and T1. The discrepancy in the number of participants included in this analysis was due to missing waist circumference data. At T0, there was no statistically significant difference in WCRF score and macronutrient intake (protein, fat, saturated fat, carbohydrate) between completers and noncompleters (*P* = .05).

#### Physical activity and fitness

There was a statistically significant improvement in the reported physical activity time from T0 to T1, with a median increase of 38.5 minutes per week (IQR = ‒27.5 to 122.5; *P* = .04). Further improvement was observed at T2, with a median increase of 140 minutes per week (IQR = 38.8-252.5; *P* < .001). Half the participants reported increased moderate-intensity physical activity after the T1 assessment. The proportion of participants who met the physical activity guidelines increased from 6% (*n* = 2) to 18% (*n* = 6) between T0 and T1 and to 41% (*n* = 13) at T2 (*P* < .001).

There was a statistically significant improvement in the 30-second chair stand after the intervention (*P* = .002) but not timed up and go or 2-minute step test (*P* = .17) between T1 and T0 (Table 4). There was an improvement in 2-minute step test (*P* = .03) between T0 and T2.

**Table 4.** Fitness assessments between T0, T1, and T2 follow-up.

Fitness assessment		**T0 vs T1** **(*n* = 33)**				**T0 vs T2** **(*n* = 31)**			
	Completers at baseline (*n* = 34)	T0	T1	**Median or Mean** **difference^c^**	*P*	T0	T2	Median or Mean difference^c^	*P*
30-s chair stand (No. of stands within 30 s), mean (SD)	12.8 (3.2)	13 (3.1)	14.4 (4.09)	Mean ‒1.4 (95% CI = ‒2.23 to –0.56)	.002^a^	13 (3.3)	15.1 (4.4)	Mean –2.2 (95% CI = –3.1 to –1.3)	<.001^a^
2-min step test (No. of steps), median (IQR)	92 (78.8-104.3)	92 (80.0-104.5)	98 (86.0-109.0)	–5 (–12.0 to 6.0)	.32^b^	90 (78.0-105.0)	100 (85.0-106.0)	–6 (–13.0 to 4.0)	.03^b^
Timed up & go,^b^ median (IQR), s	5.9 (4.8-7.3)	5.9 (5.5-7.2)	6.2 (5.5-7.4)	–0.27 (–0.8 to 5)	.17^b^	(*n* = 32) 5.9 (5.5-7.3)	(*n* = 32) 6.51 (5.6-7.3)	–0.35 (–0.9 to 0.4)	.14^b^

Abbreviations: T0 = baseline; T1 = immediately after intervention; T2 = 3 months after intervention.

a Paired *t* test for paired data between T0 and T1, T0 and T2; 2-sided *P* value.

b Tests of normality: Shapiro-Wilk *P* < .05; nonparametric test; related samples Wilcoxon signed rank test was used for comparison purposes.

c Median unless otherwise specified.

#### Anthropometry

There was no statistically significant difference in body weight or BMI between T0 and T1 (*P* > .05), but there was a statistically significant increase in weight (1 kg [IQR = ‒0.9 to 3.4], *P* = .02) and BMI (0.36 [IQR = ‒0.3 to 1.2], *P* = .02) at T2. Participants with waist circumference data available (*n* = 30) showed a 1.9-cm (95% CI = 0.40 to 3.34) reduction in waist circumference at T1 and a 3.29-cm reduction (95% CI = 1.88 to 4.70) at T2 (Table 5).

**Table 5.** Comparisons of anthropometric measurements between T0, T1, and T2 assessments

	**Completers, T0** **(*n* = 34)**	**T0** **(*n* = 32)**	**T1^a^** **(*n* = 32)**	**Median or Mean** **difference**	*P*	**T0** **(*n* = 32)**	T2^a^ (*n* = 32)	Median or Mean difference^d^	*P*
Body weight, median (IQR), kg	70.3 (60.8-83.8)	68.5 (60.3-82.3)	70.3 (57.9-82.6)	0.9 (‒0.5 to 1.0)	.20^b^	70.25 (60.3-82.3)	69.75 (59.0-84.5)	1.0 (‒0.9 to 3.4)	.02^b^
BMI, median (IQR), kg/m^2^	25.8 (22.4-29.8)	24.9 (22.2-29.6)	24.9 (21.1-29.5)	0.30 (‒0.5 to 1.0)	.22^b^	25.8 (22.2-30.0)	26.4 (20.7-29.3)	0.36 (‒0.3 to 1.2)	.02^b^
Waist circumference, cm	*n* = 32	*n* = 30	*n* = 30		.01^c^	*n* = 30	*n* = 30		<.001^c^
	Mean (SD) = 87.3 (14.7)	Mean (SD) = 87.6 (14.68)	Mean (SD) = 85.8 (14.17	Mean = 1.9 (95% CI = 0.40 to 3.34)		Mean (SD) = 88.6 (14.7) [95% CI = 65.0 to 120.0]	Mean (SD) = 85.3 (14.4) [95% CI = 62.0 to 115.0]	Mean = 3.3 (95% CI = 1.88 to 4.70]	

Abbreviations: BMI = body mass index; T0 = baseline; T1 = immediately after intervention; T2 = 3 months after intervention.

a Paired data between T0 and T1 (*n* = 32) and T0 and T2 (*n* = 32).

b Tests of normality: Shapiro-Wilk *P* < .05; for nonparametric test related samples Wilcoxon signed rank test was used for comparison purposes.

c Paired *t* test, 2-sided *P* values.

d Median unless otherwise specified.

#### Quality of life

Quality of life among completers and noncompleters was similar in the summary, global health, functional, and symptom scales (*P* > .05) (data not shown). The median summary score for the 34 completers was 80.9 (IQR = 67.4-89.2) at T0, but that value decreased at T1 (73.6 [IQR = 63.1-84.1]) before improving at T2 (85.1 [IQR = 75.8-89.9]), although compared with baseline, the changes were not statistically significant (*P* > .05). There were substantial differences in some domain scores and symptoms, such as lower physical functioning, social functioning, sexual functioning, and body image functional scales, and higher symptoms, such as fatigue, diarrhea, systemic therapy side effects, breast symptoms, and financial difficulties, at T1 (Table S1).

**Figure 3.**
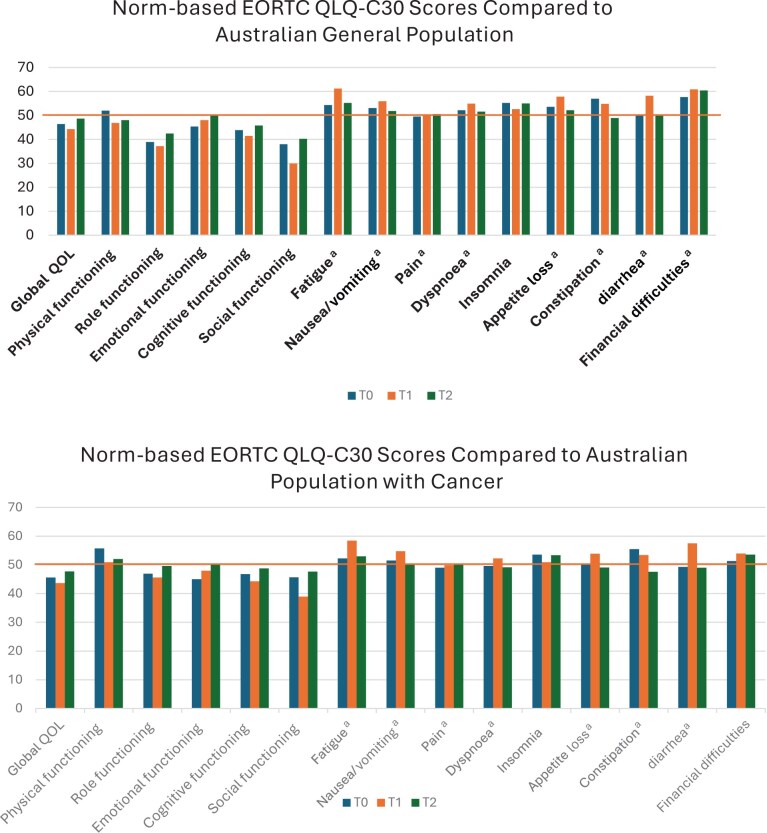
**(A)** Comparisons of QOL scores between study participants and the Australian general population at baseline, immediate postintervention, and 3-month postintervention follow-up. **(B)** Comparisons of QOL scores between study participants and the Australian general population at baseline, immediate postintervention, and 3-month postintervention follow-up.^33^ Abbreviations: EORTC = European Organisation for Research and Treatment of Cancer; QLQ-C30 = QLG Core Questionnaire; QOL = quality of life; T0 = baseline; T1 = immediately after intervention; T2 = 3 months after intervention. Orange horizontal line represents Australian population mean. Higher score in QOL indicates better QOL, and higher functioning scales indicate better functioning. ^a^Higher symptom scores indicate worse symptoms.

Compared with the general Australian population, study participants scored lower on role functioning and social functioning and higher on symptom scores, indicating poorer QOL. Study participants reported similar physical functioning but lower scores on role functioning, cognition, and social functioning (Figure 3, A and B) compared with the Australian cancer population.

The median distress level was higher at T0 than at T1 (6 [IQR = 2-7] vs 3 [IQR = 1-6]; *P* = .08) and at T2 (3 [IQR = 1-5.8]). The proportion of participants with distress levels rated as a 4 or above was 62% at T0, 45.5% at T1, and 37.5% at T2—not statistically significantly different from baseline.

## Discussion

This study used a pragmatic, choice-based approach, allowing participants to set their own dietary and exercise goals after considering national recommendations.^5^^,^^28^ We assessed the feasibility of the LEAD-4-BCS intervention to promote healthy eating and physical activity during chemotherapy. Setting achievable and measurable dietary and exercise goals, with a supporting intervention that can lead to an improvement in diet or physical activity, as illustrated in the study results (eg, an improvement in WCRF score, higher proportion of participants meeting recommended fruit and vegetable servings, and higher median time spent on physical activity), is important in a lifestyle intervention trial. If the LEAD-4-BCS intervention was able to support 60% of the study participants in reaching their preset dietary or exercise goals, it suggests that such an intervention is feasible for promoting a healthy lifestyle during chemotherapy, a challenging period for cancer survivors. One-third of participants achieved at least 50% of their self-determined dietary and exercise goals initially—lower than expected. However, 3 months after the intervention, the proportion achieving at least 50% of their preset dietary and exercise goals increased to 65%, indicating that a 12-week structured diet counselling and exercise physiologist–supervised exercise intervention, delivered virtually, is feasible for breast cancer survivors undergoing chemotherapy. A longer follow-up period is required to assess further behavioral improvement or maintenance of dietary or exercise goals.^37^

Other lifestyle intervention studies have shown variable adherence. The Impact of Nutritional Intervention in Women With Breast Cancer Under Adjuvant Chemotherapy (PASAPAS; ClinicalTrials.gov identifier NCT01331772) randomized controlled trial of exercise and nutritional interventions in breast cancer survivors receiving adjuvant chemotherapy found that only 10% were fully compliant with the study intervention. Based on attendance at supervised and unsupervised exercise sessions during the 6-month study period, 54% complied with 70% of the intervention.^22^ This finding is comparable to our study, with 44% attendance for exercise sessions and 75% attendance for both the dietary and supervised exercise sessions. The multicenter Effect of a Program of Physical Activity and Nutritional Therapeutic Education in Breast Cancer Patients (APAD2; ClinicalTrials.gov identifier NCT04109326) randomized controlled trial,^38^ incorporating an exercise and diet counseling intervention for breast cancer survivors during adjuvant treatment, found 65.5% adherence to both diet and exercise programs, with rates of 80% for the intervention arm and 88% for the control arm over 26 weeks. Higher adherence in APAD2 may be attributed to face-to-face delivery and the longer duration (26 weeks vs our 12 weeks). Another US-based randomized controlled trial of both exercise and dietary interventions in 38 breast cancer survivors during neoadjuvant chemotherapy reported 100% retention; however, some missed study assessments (*n* = 5 midchemotherapy, *n* = 10 after chemotherapy). The average attendance for the intervention group was 80% for face-to-face sessions, dropping to 43% when sessions were delivered by telephone. The authors suggested that these differences could be due to treatment schedules. In our study, some participants lost contact during the intervention period. Without a researcher on-site, we were unable to determine whether external factors such as hospitalizations, treatment delays, or rescheduled surgery affected adherence.

Our participants demonstrated a slight increase in weight. This finding was not unexpected because our intervention aimed to improve (or maintain) diet and exercise rather than weight management. A substantial reduction in waist circumference was observed, which may have represented a favorable body composition change. Although we provided careful instructions for waist self-assessment, self-measured waist circumference has been shown to have high intraobserver error.^39^ Similar findings of no weight loss were seen in the PASAPAS^22^ and Weight Gain Prevention for Breast Cancer Survivors (BALANCE; ClinicalTrials.gov identifier NCT00533338) studies, with a small reduction in waist circumference reported in BALANCE.^40^

Physical activity levels improved over time, most noticeably 3 months after the intervention. This finding suggests that participants may require longer (>12 weeks) to improve their physical activity while on chemotherapy. Their treatment schedule may have also affected this rate, as 50% were receiving neoadjuvant treatment during the intervention and treatment may have changed soon after T1 (from chemotherapy to radiation therapy or surgery). Unfortunately, we did not collect detailed treatment data, limiting our ability to explore these patterns.

Our participants had lower global QOL scores and functional scales, except physical functioning, compared with the Australian general and cancer populations. Global QOL improved over time but remained lower. The functional scales most affected were role and social functioning, both 1 SD lower than the general population. These results and our qualitative data highlight the impact of cancer and its treatment on social functioning during treatment, emphasizing the importance of social support. We incorporated brief peer support into the dietary education sessions, encouraging participants to share their experience and questions. It is unclear whether this influenced participants’ emotional and social well-being or the increased attendance at diet education sessions compared with exercise sessions.

We used a pragmatic intervention approach for this feasibility study that required minimal staff resources. It realistically represents the current Australian healthcare setting. Recruitment was feasible, with high acceptance by breast cancer survivors. Noncompliance or early withdrawal for medical reasons was similar to other lifestyle intervention studies (eg, PASAPAS).^22^ Despite some studies showing better adherence with face-to-face models, our results suggest that a telehealth model can achieve desirable adherence once participants have sufficient capacity to build healthy lifestyle behaviors. Maintaining regular contact with service providers and providing further reminders may be required. Moreover, the intervention offers the potential to expand access to this behavior change modality to breast cancer survivors unable to access on-site exercise programs because of distance, availability, or social barriers.

### Study limitations and strengths

All data except physical fitness assessment were self-reported, so caution is needed when interpreting some outcomes, particularly medical information and anthropometry, because social desirability, recall, and response bias associated with self-reporting cannot be excluded. This study did not explore the impact of menopausal status on overall QOL and body composition, especially relevant to breast cancer survivors who experienced chemotherapy-induced menopause.^41^ Further exploration of preferences, enablers, and barriers to adherence to the intervention is needed to support participants in the planned phase 3 trial.

Participants with lower computer literacy or internet connection issues are less likely to engage in online interventions. A systematic review identified issues such as individual intrinsic factors (eg, lack of confidence, self-efficacy), technological availability, difficulties accessing and using e-health, and insufficient technological support that influence older (e.g, 60 years or older) adults engaging in e-health.^42^ To help ensure that computer literacy was not a barrier, our current study incorporated simple technology support, such as help for participants setting up their connection (eg, written instructions or telephone support from the study coordinator); using common platforms such as Zoom; and participants being able to use smartphones, tablets, or computers. Since the COVID-19 pandemic, many people are more familiar with e-health services and virtual platforms, which may have facilitated participation. One of the preferences for physical exercise and activity programs commonly noted among breast cancer survivors was group support settings, which include peer and professional support and the opportunity to network and receive social support, with accountability serving as their motivation.^43^ We used a virtual platform for dietary and exercise sessions in a group setting where participants received peer and professional support in a comfortable and familiar environment. Participants were asked to keep a diary of their home-based exercise and fruit and vegetable intake to encourage self-awareness and accountability. The study was feasible for participants from remote areas or areas with limited dietitian and exercise physiologist services. From an operational perspective, it is possible to offer such interventions through a virtual platform using a centralized, experienced team (dietitian and exercise physiologist 2 days/week).

Using a virtual platform to deliver a structured, supervised exercise and diet program to breast cancer survivors during adjuvant or neoadjuvant chemotherapy is feasible, but the primary adherence endpoint was not met immediately after the intervention, although it was met 3 months after the intervention, suggesting that a longer study intervention period may be needed to support breast cancer survivors through completion of chemotherapy. The intervention was acceptable, and the proportion of participants who met dietary guidelines for fruit and vegetables as well as exercise continued to improve after the intervention period. This finding indicates the need for further investigation on a larger scale, with some study design modifications to improve participant engagement.

## Supplementary Material

pkaf122_Supplementary_Data

## Data Availability

Data are available on reasonable request from the author.
