# Supplementary material for: Feasibility evaluation of a virtual lifestyle intervention for early-stage breast cancer survivors undergoing chemotherapy
Source: JNCI Cancer Spectr. 2026 Jan 31;10(1):pkaf122. doi: 10.1093/jncics/pkaf122 (PMC12909259; doi:10.1093/jncics/pkaf122)
Supplement: pkaf122_Supplementary_Data [file pkaf122_supplementary_data.docx]

# **Supplementary Material**

# **Supplementary Methods:**

**Secondary outcomes:**

**Acceptability**

Acceptability was measured through participation rate, attendance rate, and qualitative interviews. Participation rate was calculated as the number of participants who consented divided by the number of eligible referrals from the study sites. The attendance rate was calculated as the number of dietary and exercise sessions attended divided by the total number of sessions offered.

Exit interview: Optional semi-structured interviews were conducted by GC when participants exited the study, including withdrawals. Interviews explored participants’ experiences with the intervention, including use of virtual platforms, and barriers and enablers to adhering to the study intervention. Full data will be reported elsewhere.

**Lifestyle outcomes**

**Anthropometry**

Participants self-reported height at T0, and body weight and waist circumference at each assessment (T0, T1, T2). Pictures and instructions on how to measure waist circumference were included in the questionnaire to assist with consistency at each assessment time point.

**Diet**

A 3-day food diary (recording all food and drink consumption over two weekdays and one weekend day, or three consecutive days if there were no changes in dietary patterns over weekends) was used to measure participants’ nutrient and fruit and vegetable intake at each assessment. Two trained dietitians conducted the analysis using FoodWorks 10 Premium Edition, v10.0.

A World Cancer Research Fund (WCRF) score was calculated according to ‘*Scoring standards for the 2018 WCRF/AICR Score*’.(28) This score reflects participants’ adherence to lifestyle recommendations for cancer prevention from the WCRF/American Institute for Cancer Research. Scores were allocated as either a full or half point based on the participants’ anthropometry, physical activity, and diet quality; a higher score indicates greater adherence.

**Exercise**

Physical fitness was assessed virtually using the 30-second chair stand, timed up-and-go (TUG), and 2-minute step test (29). Reported physical activity was collected using the modified Godin Leisure-Time Exercise Questionnaire (mGLTEQ) (30), capturing frequency and duration of light, moderate and vigorous-intensity aerobic and resistance training in the past 7 days.

**Quality of life (QoL)**

Participants completed the 30-item European Organisation for Research and Treatment of Cancer (EORTC) Quality of Life of Cancer patients (QLQ-C30) and the 23-item breast cancer module (QLQ-BR23) (31, 32) at T0, T1 and T2. Higher scores in each functioning scale indicate lower disease and treatment impact on QoL; higher symptom scores indicate higher symptom burden. Calculation of the QoL scores from EORTC QLQ-C30 and BR-23 were based on the scoring manual. (33)

A single-item distress thermometer (34) was used to assess participants' psychological distress levels over the previous seven days.

***SMS messages sent to study participants***

Examples of messages that were well received in the study ‘Likeability and perceived effectiveness of messages designed to encourage physical activity participation among older adults’ by Jongenelis, et al. 2022(23)

- *Be active 30-60min a day to stay fit and well*
- *Use it or lose it*
- *This is your time – enjoy being strong and active*
- *Let us take it on, one step at a time*
- *Move more, live longer*
- *Stay fit to stay functional*

Additional questions prompting participants to exercise and to adhere to a healthy eating pattern

- Have you done any exercise this week?
- It is not too late to get more steps in if you haven’t achieved your goal. If you have achieved your exercise goal – well done!
- Have you eaten your 2 pieces of fruit today?
- Vegetables such as cherry tomatoes, baby cucumbers, celery, carrots can be a snack too.
- Feeling tired? Studies show some exercise can help with fatigue
- A short 10 minute walk is better than none
- Craving snacks? Healthier options include fruit, low fat yoghurt, and crunchy vegetables such as carrots, celery sticks, baby cucumbers

**Table S1: Quality of life measured by EORTC QLQ C-30 and BR-23 (median, interquartile range and range) for study participants at baseline (T0), post-intervention (T1) and 3-month follow-up (T2) assessments.**

|  | T0  N=34 | T1  N=32  Missing n=2 | P value (T1-T0) | T2  N=32  Missing n=2 | P value  (T2-T0) |
| --- | --- | --- | --- | --- | --- |
|  | Median (IQR)  (Range) | Median (IQR)  (Range) |  | Median (IQR)  (Range) |  |
| EORTC QLQ C-30 | | | | | |
| Summary Score (13 items) | 80.9 (21.8)  (48.9 to 97.4) | 73.6 ( 20.94)  (44.1 to 93.1) | 0.062 | 85.1 (14.1)  (35.0 to 96.2) | 0.270 |
| Global health status (QoL) | 62.5 (33.3)  (16.7 to 91.7) | 66.7 (16.7)  (8.3 to 83.3) | 0.188 | 66.7 (25.0)  (25.0 to 100.0) | 0.403 |
| Physical Functioning (PF2) | 100 (6.67)  (66.7 to 100) | 93.3 (18.3)  (60 to 100) | <0.001^a^ | 93.3 (18.3)  (53.3 to 100.0) | 0.035^a^ |
| Role Functioning (RF2) | 66.7 (50.0)  (0 to 100) | 66.7 (33.3)  (0 to 100) | 0.481 | 83.3 (33.3)  (0 to 100) | 0.226 |
| Emotional Functioning (EF) | 75.0 (35.42)  (8.3 to 100) | 75.0 (25.0)  (0 to 100) | 0.104 | 83.3 (25.0)  (16.7 to 100.0) | 0.015 |
| Cognitive Functioning (CF) | 83.3 (33.3)  (16.7 to 100) | 66.7 (16.7)  (0 to 100) | 0.07 | 83.3 (33.3)  (0 to 100) | 0.824^a^ |
| Social Functioning (SF) | 75.0 (33.3)  (0 to 100.0) | 50.0 (33.3)  (0 to 100) | 0.01 | 66.7 (33.3)  (16.7 to 100) | 0.839^a^ |
| Fatigue (FA) | 33.3 (44.4)  (0 to 100) | 44.4 (33.3)  (11.1 to 100.0) | 0.005 | 33.3 (30.6)  (0.0 to 77.8) | 0.215 |
| Nausea and Vomiting (NV) | 0.0 (16.7)  (0 to 33.3) | 8.3 (16.7)  (0 to 50.0) | 0.197 | 0.0 (16.67) (0.0 to 50.0) | 0.308 |
| Pain (PA) | 16.7 (33.3)  (0 to 66.7) | 16.7 (33.3)  (0 to 66.7) | 0.734 | 16.7 (33.3)  (0 to 66.7) | 0.664 |
| Dyspnoea (DY) | 0.0 (33.3)  (0 to 66.7) | 16.7 (33.3)  (0.0 to 66.7) | 0.134 | 0.0 (33.3)  (0 to 66.7) | 1.00 |
| Insomnia (SL) | 33.3 (66.7)  (0 to 100) | 33.3 (58.3)  (0 to 66.7) | 0.354 | 33.3 (58.3)  (0.0 to 100.0) | 0.680 |
| Appetite Loss (AP) | 0 (33.3)  (0 to 100) | 16.7 (33.3)  (0 to 100.0) | 0.097 | 0.0 (33.3)  (0 to 66.7) | 1.00 |
| Constipation (CO) | 0 (33.3)  (0 to 100) | 0.0 (33.3)  (0 to 66.7) | 0.292 | 0.0 (0.0)  (0 to 100) | 0.002^a^ |
| Diarrhoea (DI) | 0.0 (0.0)  (0 to 66.7) | 0.0 (33.3)  (0.0 to 100.0) | 0.013 | 0.0 (0.0)  (0.0 to 66.7) | 1.00^a^ |
| Financial Difficulties (FI) | 0.0 (33.3)  (0 to 100) | 33.3 (66.7)  (0.0 to 100.0) | 0.019 | 16.7 (33.3)  (0 to 100) | 0.039 |
| Breast specific module (BR-23) | | | | | |
| Systemic therapy side effects (ST) | 9.5 (20.2)  (0 to 47.6) | 38.1 (23.8)  (0.0 to 81) | <0.001 | 11.9 (18.9)  (0.0 to 57.1) | 0.177 |
| Upset by hair loss (HL)  (n=13) | 66.7 (33.3)  (33.3 to 100.0) | 33.3 (33.3)  (0.0 to 100.0) | N=6  0.180 | 33.3 (75.0)  (0 to 100) | N=5  0.625^a^ |
| Arm symptoms (AS) | 0.0 (22.2)  (0 to 55.6) | 11.1 (19.4)  (0.0 to 100.0) | 0.652 | 11.1 (33.3)  (0 to 77.8) | 0.120 |
| Breast Symptoms (BS) | 16.7 (29.2)  (1 to 100) | 8.3 (16.7)  (0.0 to 100.0) | 0.001 | 16.7 (25.0)  (0 to 100) | 0.690^a^ |
| Body Image (BI) | 83.3 (33.3)  (0 to 100) | 62.5 (41.7)  (0.0 to 100.0) | <0.001^a^ | 66.7 (39.6)  (0 to 100) | 0.007^a^ |
| Future Perspective (FU) | 50.0 (41.7)  (0 to 100.0) | 33.3 (58.3)  (0.0 to 100.0) | 0.311 | 33.3 (66.7)  (0 to 100.0) | 0.152 |
| Sexual Functioning (SEF) | 16.7 (33.3)  (0 to 66.7) | 0.0 (66.7)  (0.0 to 66.7) | 0.013^a^ | 0 (29.2)  (0 to 66.7) | 0.032 |
| Sexual Enjoyment (SEE)  (n=23) | 66.7 (33.3)  (33.3 to 100.0) | 33.3 (33.3)  (0.0 to 100.0) | N=8  0.070^a^ | 33.3 (66.7)  (0 to 100) | N=8  0.063^a^ |

All results are presented as median (IQR) (range) unless otherwise specified
